# Utilization of non-invasive measures to evaluate eccentric exercise damage in an untrained population

**DOI:** 10.1186/1550-2783-11-S1-P37

**Published:** 2014-12-01

**Authors:** Parker Hyde, Ciaran Fairman, Josh Beck, Keagan Kiely, Nicholas Coker, Mary-Beth Yarbrough, Kylie Phillips, Kristina Kendall

**Affiliations:** 1 Georgia Southern University, Statesboro, Georgia, USA

## Background

One of the most common modes of exercise among males is resistance training. The associated movements among the most common resistance training exercises completed include periods of both concentric and eccentric contraction. Previous research has demonstrated that repeated eccentric contractions of a muscle produce damaging results to the muscles involved. Invasive measures, such as blood draws and muscle biopsies have been utilized to assess skeletal muscle damage in individuals. However, the efficacy of the utilization of Creatine Kinase concentration as a marker of recovery has recently been called into question. Furthermore, biopsies and blood draws may not be applicable to identify muscle damage and recovery in an untrained population for the nutritional or strength and conditioning professional

## Methods

Subjects (n= 19) volunteered to participate in this study and were untrained (resistance training) male college students between the ages of 18-30. Untrained criteria consisted of less than two years lower body resistance training. Prior to completion of the study participants completed a health history questionnaire. Participants were asked to refrain from physical activity for the duration of the study. The first day of testing included completion of a muscle soreness scale (DOMS), range of motion (ROM) of the dominant knee, swelling of the vastus lateralis (SWVL) via ultrasound (Terason T3200), Peak force measurement using an isokinetic dynamometer, a fatiguing exercise protocol on the dynamometer (a total of 50 eccentric contractions set at 120% of peak MVC force with an angular velocity of 60°/sec), followed immediately by post-test measurements of DOMS and peak force. The peak force measurement was determined using an isometric maximum voluntary contraction (MVC) of the quadriceps on the dynamometer with the knee joint held at 45°. The participants were then asked to return to the lab 24, 48 and 72 hours later. Each returning visit included a battery of tests comprising of measurements of DOMS, ROM, SWVL-Terascape, SWVL-Long, SWVL-Trans and peak force. Repeated measures ANOVAs (placebo vs. multi ingredient supplement) were used to analyze changes in peak power, intramuscular swelling, DOMS, and ROM. The alpha level was set at p≤ 0.05. Consent to publish the results was obtained from all participants.

## Results

Mean scores of DOMS for all participants differed significantly between baseline and immediate post (p=.001), 24 (p=.007), 48 (p=.001), 72-hours (p=.004) respectively. Also, significant differences for DOMS were found between baseline, post (p=0.001), 24 (p=.007), 48 (p=.001), and 72-hours (p=.004). Further significant differences were observed between 24 and 48-hours (p=0.008), 48 and 72-hours (p=0.008). Mean scores for PForce differed significantly between baseline and immediate post (p<.001), 24 (p=.003), and 72-hours (p=.002). Mean scores between baseline and 48-hours were just barely non-significant (p=.06). ROM means were significantly different between 48-h and 72-hours (p=.009). SWVL-Long had a significant effect of time between pre-fatiguing and 72-hours (p=.045). No significant effects of time were found on the SWVL-Terascape measurement or SWVL-Trans.

## Conclusion

We conclude that certain noninvasive measures can be utilized to determine muscle damage in an untrained population. This study further exemplified the importance of strength measurement being one of the key components of skeletal muscle damage. The utilization of panoramic or transverse ultrasound imaging to assess skeletal muscle swelling in an untrained population following eccentric exercise may not be warranted. Due to the small sample size, the main effect of time may not be able to be observed so future studies evaluating non-invasive measures of assessment may seek for a greater sample. Additionally, DOMS may be a reliable predictor of physiological performance and recovery in untrained male participants.

